# Impact of enhanced recovery after surgery protocol compliance on patients’ outcome in benign hysterectomy and establishment of a predictive nomogram model

**DOI:** 10.1186/s12871-021-01509-0

**Published:** 2021-11-22

**Authors:** Yiwei Shen, Feng Lv, Su Min, Gangming Wu, Juying Jin, Yao Gong, Jian Yu, Peipei Qin, Ying Zhang

**Affiliations:** 1 Department of Anesthesiology, The First Affiliated Hospital of Chongqing Medical University, No.1 Youyi Road, Yuzhong District, Chongqing, 400016 People’s Republic of China; 2 Department of Gynecology, the First Affiliated Hospital of Chongqing Medical University, Chongqing, China

**Keywords:** Enhanced recovery after surgery (ERAS), Hysterectomy, Compliance, Postoperative complications, Patient satisfaction, Patient experience

## Abstract

**Background:**

Enhanced recovery after surgery (ERAS) pathways have been shown to improve clinical outcomes after surgery. Considering the importance of patient experience for patients with benign surgery, this study evaluated whether improved compliance with ERAS protocol modified for gynecological surgery which recommended by the ERAS Society is associated with better clinical outcomes and patient experience, and to determine the influence of compliance with each ERAS element on patients’ outcome after benign hysterectomy.

**Methods:**

A prospective observational study was performed on the women who underwent hysterectomy between 2019 and 2020. A total of 475 women greater 18 years old were classified into three groups according to their per cent compliance with ERAS protocols: Group I: < 60% (148 cases); Group II:≥60 and < 80% (160 cases); Group III: ≥80% (167 cases). Primary outcome was the 30-day postoperative complications. Second outcomes included QoR-15 questionnaire scores, patient satisfaction on a scale from 1 to 7, and length of stay after operation. After multivariable binary logistic regression analyse, a nomogram model was established to predict the incidence of having a postoperative complication with individual ERAS element compliance.

**Results:**

The study enrolled 585 patients, and 475 completed the follow-up assessment. Patients with compliance over 80% had a significant reduction in postoperative complications (20.4% vs 41.2% vs 38.1%, *P* < 0.001) and length of stay after surgery (4 vs 5 vs 4, *P* < 0.001). Increased compliance was also associated with higher patient satisfaction and QoR-15 scores (*P* < 0.001),. Among the five dimensions of the QoR-15, physical comfort (*P* < 0.05), physical independence (*P* < 0.05), and pain dimension (*P* < 0.05) were better in the higher compliance groups. Minimally invasive surgery (MIS) (*P* < 0.001), postoperative nausea and vomiting (PONV) prophylaxis (*P* < 0.001), early mobilization (*P* = 0.031), early oral nutrition (*P* = 0.012), and early removal of urinary drainage (*P* < 0.001) were significantly associated with less complications. Having a postoperative complication was better predicted by the proposed nomogram model with high AUC value (0.906) and sensitivity (0.948) in the cohort.

**Conclusions:**

Improved compliance with the ERAS protocol was associated with improved recovery and better patient experience undergoing hysterectomy. MIS, PONV prophylaxis, early mobilization, early oral intake, and early removal of urinary drainage were of concern in reducing postoperative complications.

**Trial registration:**

Chinese Clinical Trial Registry, ChiCTR1800019178. Registered on 30/10/2018.

**Supplementary Information:**

The online version contains supplementary material available at 10.1186/s12871-021-01509-0.

## Background

Enhanced recovery after surgery (ERAS) pathway is a multimodal perioperative care approach, designed to reduce perioperative stress and shorten recovery time [1]. The success of the ERAS pathway is attributed to the synergy between its elements [2, 3]. Although ERAS protocols comprise several different perioperative interventions, not every participant is able to complete the protocols. Implementation and adherence to the protocol is crucial to achieve, [4–6] with the challenges including lack of knowledge, resistance to change, and shortage of staff [7]. A growing number of researches have confirmed that the compliance with ERAS protocols is associated with postoperative rehabilitation in patients undergoing colorectal, orthopedic and other surgeries [8, 9].

Hysterectomy on benign indication is the most common major gynecological surgery performed worldwide [10]. The aim of surgery for benign diseases is primarily to improve the quality of life related to health, and high-quality recovery and good patient experience after surgery are therefore very important for both patients and society. Patient-reported outcomes are the patient-centered way to track and study all stages of surgical recovery across multiple domains and longitudinal time span [11].

Recently, enhanced recovery protocols have been introduced in general settings for gynecological surgeries [12–15]. However, to date, no clinical studies have focused on ERAS protocol compliance for benign hysterectomy, particularly patient self-assessment and satisfaction. This study aims to evaluate whether improved compliance with ERAS protocol modified for gynecological surgery is associated with better clinical outcomes and patient experience, and to determine the impact of compliance with each ERAS element on patient outcomes after hysterectomy. In addition, we developed a model to predict the incidence of having a postoperative complication with individual ERAS element compliance.

## Methods

This study was approved and conducted by the local Ethics Committee of the First Affiliated Hospital of Chongqing Medical University (No.2019**–**020), and registered with ClinicalTrials.gov (ChiCTR1800019178, 30/10/2018). Patients were informed of the ERAS protocols and signed the informed consent form before study entry, and the study was conducted in accordance with the Declaration of Helsinki. A prospective observational study on patients undergoing benign hysterectomy was performed at the Department of Gynecology in the First Affiliated Hospital of Chongqing Medical University in China during the period February 1st 2019 to December 31st 2020, with the ERAS protocols underwent.

### Study design

Inclusion criteria for the subjects were age more than 18 years old with elective open or laparoscopic hysterectomy for benign conditions. Exclusion criteria were genital prolapse as indication for the hysterectomy, previous bilateral oophorectomy or the present operation would leave the woman without ovaries, physically or mentally disabled, severe psychiatric disease, or informed consent could not be obtained. All hysterectomies were total, but the type of surgical procedure was up to the operating surgeon. The criteria for laparoscopy or laparotomy were based on the Clinical guidelines for treatment [16, 17]. To avoid the influence of radiotherapy or chemotherapy on patients experience and outcomes, patients with malignant and borderline tumors were excluded. Six surgeons, twelve nurses, five anaesthetists, one dieticians, and three physiotherapists formed the ERAS multi-disciplinary team (MDT), which effectively implemented the ERAS protocols. The protocols are based on the practice guidelines for gynecologic surgery by the ERAS society [16**–**18] and consist of 22 items involving preoperative, intraoperative and postoperative interventions (Additional file 1). All treatments were performed by ERAS MDT. To ensure that the protocols were running smoothly, everyone on the team communicated with each other and reported their work at weekly meetings. The study protocol conforms to GCP (Good Clinical Practice) standard procedures, and all investigators were trained and certified.

The Department of Gynecology of the First Affiliated Hospital of Chongqing Medical University serves a large geographic area in western and southwestern China with a referral base of 2 million people. The first gynecological oncology guidelines recommended by the ERAS Society were published in 2016 [18, 19] and revised in 2019 [20], which was formally carried out in our hospital from October in 2016. Every patients would receive a “rehabilitation log” which containing perioperative ERAS guidelines and survey on each items. Periodic text message reminders were sent to remind participants about completion of the rehabilitation logs every day, such as early mobilization and early oral intake. For example, on the first postoperative day, in addition to the pre-operative education by nurses, mobile phone messages would remind patients that they should have an out-of-bed activity for 2 **h**.

The implementation of each item for each patient and outcome were collected prospectively. For categorical elements compliance was marked as yes/no. For intravenous fluids on the first postoperative day, ERAS compliance with balanced fluids was set to less than 2000 ml. The compliance rate for each patient was calculated as the number of interventions fulfilled/22 (total number of ERAS items). Mean total compliance was calculated as the average of all perioperative ERAS interventions. Patients were categorized into three groups by their compliance rates with the ERAS pathway. The same cut-off values as previous studies were used, [21, 22] where compliance was classified as ‘poor’, ‘partial’, or ‘full’ when **<** 50%, ≥50%, or ≥ 80%. However, in the pilot trial before the formal start of this study, only a very small number of patients had a compliance rate < 50%, so the poor group was classified as **<** 60%. Each patient was followed by the ERAS team members during the hospital stay.

Characteristics of the study population, age, body mass index (BMI), education level, smoking status, nutritional risk screening (NRS) score, NYHA status, ASA status, history of surgery, preoperative hemoglobin, presence of chronic pain, history of postoperative nausea and vomiting **(**PONV), diagnosis and type of surgical procedure were recorded. Preoperative comorbidities were also recorded, such as hypertension, diabetes, chronic obstructive pulmonary disease (COPD), ischemic heart disease and asthma. After entering the operating room, the plasma electrolyte level was monitored by invasive arterial pressure puncture. Preoperative hypokalemia was defined as less than 3.5 mmol/L. Intraoperative data were prospectively collected, including duration of surgery (min), intraoperative net fluid input and blood loss.

All of the patients were asked to complete 15-item quality of recovery (QoR-15) questionnaire, a widely used self-rated questionnaire for early postoperative quality of recovery [23, 24] (Additional file 2), under the guidance of the investigator on the day before operation, then to repeat it 24 h/48 h/72 h postoperatively [25]. QoR-15 is a patient-centered comprehensive questionnaire (15-items), which includes five aspects: physical comfort (5-items), emotional status (4-items), physical independence (2-items), psychological support (2-items) and pain (2-items) [26]. Total scores of the QoR-15 ranges from 0 to 150, higher score indicates better recovery. At the same time, their resting pain was also measured with an 11-pointed visual analogue scale (VAS) ranging from 0 to 10, with 0 indicating no pain and 10 indicating the worst pain imaginable. VAS ≥ 4 was considered to identify patients with postoperative pain of moderate-to-severe intensity. The Likert scale (strongly dissatisfied = 1, moderately dissatisfied = 2, slightly dissatisfied = 3, neutral = 4, slightly satisfied = 5, moderately satisfied = 6, extremely satisfied = 7) was used to evaluate patient satisfaction on the day of discharge and 30 days after discharge [27]. On this instrument, a higher score indicates a higher level of satisfaction.

Patients were discharged when they met the strict criteria: mobilization with normal diet, oral analgesics for pain relief, normal urination and no intestinal obstruction signs. Patients were contacted by a dedicated nurse on the telephone at 30 days after discharge. The readmission, postoperative chronic complications and patient satisfaction were also collected.

### Study outcomes

Primary outcome of the study was the incidence of postoperative complications, including PONV, moderate-to-severe postoperative pain, deep vein thrombosis (DVT), surgical site infection, and pulmonary infection. Postoperative complications were monitored until 30 days after surgery, which were defined by the guidelines for European perioperative clinical outcome definitions [28], as shown in Additional file 3.

Secondary outcomes included patient satisfaction, QoR-15 scores (including five dimensions), LOS after surgery, mortality, postoperative hospitalization costs, readmission rate within 30 days post-discharge. LOS after surgery was defined as the number of days patients stayed in the hospital after operation. Postoperative hospital costs were presented as RMB converted to Euro (November 2020), which were obtained from hospital databases.

### Statistical analysis

The characteristics of study participants in the three groups were described with a descriptive analysis, while the normal distribution of the data was checked with Shapiro-Wilk test. Outliers were investigated and eliminated in the event of a demonstrably incorrect measurement or input error. Categorical variables were described as numbers with percentages, and continuous variables were described as the means with standard deviations (SD) or median and interquartile range (IQR), depending on the distribution, which was checked through visual inspection of the histogram. The Chi square test, repeated measures analysis of variance, Mann-Whitney U test and Kruskal-Wallis test were used to compare the outcomes between groups where appropriate. Fisher’s exact test was used for categorical variables when the number of events was less than five.

Independent of the previous analysis, patients were divided into two groups based on the presence or absence of postoperative complications. All the factors including the clinicopathological factors and each component of ERAS were put into the univariable logistic regression to analyze their correlation with the probability of having any postoperative complication. Then the factors with *P* value less than 0.05 were included in the multivariable binary logistic regression and hazard ratios of each factors were calculated. Through the multivariable binary logistic regression analysis, the factors with *P* value less than 0.05 were selected to develop a nomogram prediction model by R software. The discrimination performance of the nomogram model was quantified with receiver operation characteristic (ROC) curve, with the value of the area under the curve (AUC) of the ROC curve between 0.5 and 1.0, and the closer the AUC value is to 1, the better discrimination performance the model has. Calibration curves were plotted to assess the calibration of the nomogram model.

A sample size calculation was based on the primary endpoint, the incidence of postoperative complications, as the previous study had reported that the incidence of postoperative complications undergoing gynecological operation was approximately 25% [2]. The requirement of minimum sample size for each group in this study was 118 participants to reach the statistical significance at two-sided 95% confidence interval and setting the power to 90%. To account for predicted dropout rate of 20%, we decided to recruit 542 patients. The sample size calculation was performed PASS 15.0 analysis program.

Statistical analyses were completed in SPSS for Windows version 23.0 (SPSS, Inc., Chicago, IL, USA) and GraphPad Prism for Windows version 8.0 (GraphPad Software, Inc., San Francisco, CA, USA). The nomogram plot, ROC curve and calibration curve were plotted using R software (version 3.6.2) with RMS, ROCR package(R Core Team, Vienna, Austria). A 2-sided *P* value **<** 0.05 was regarded as statistically significant.

## Results

### Patient characteristics

**Fig. 1:**
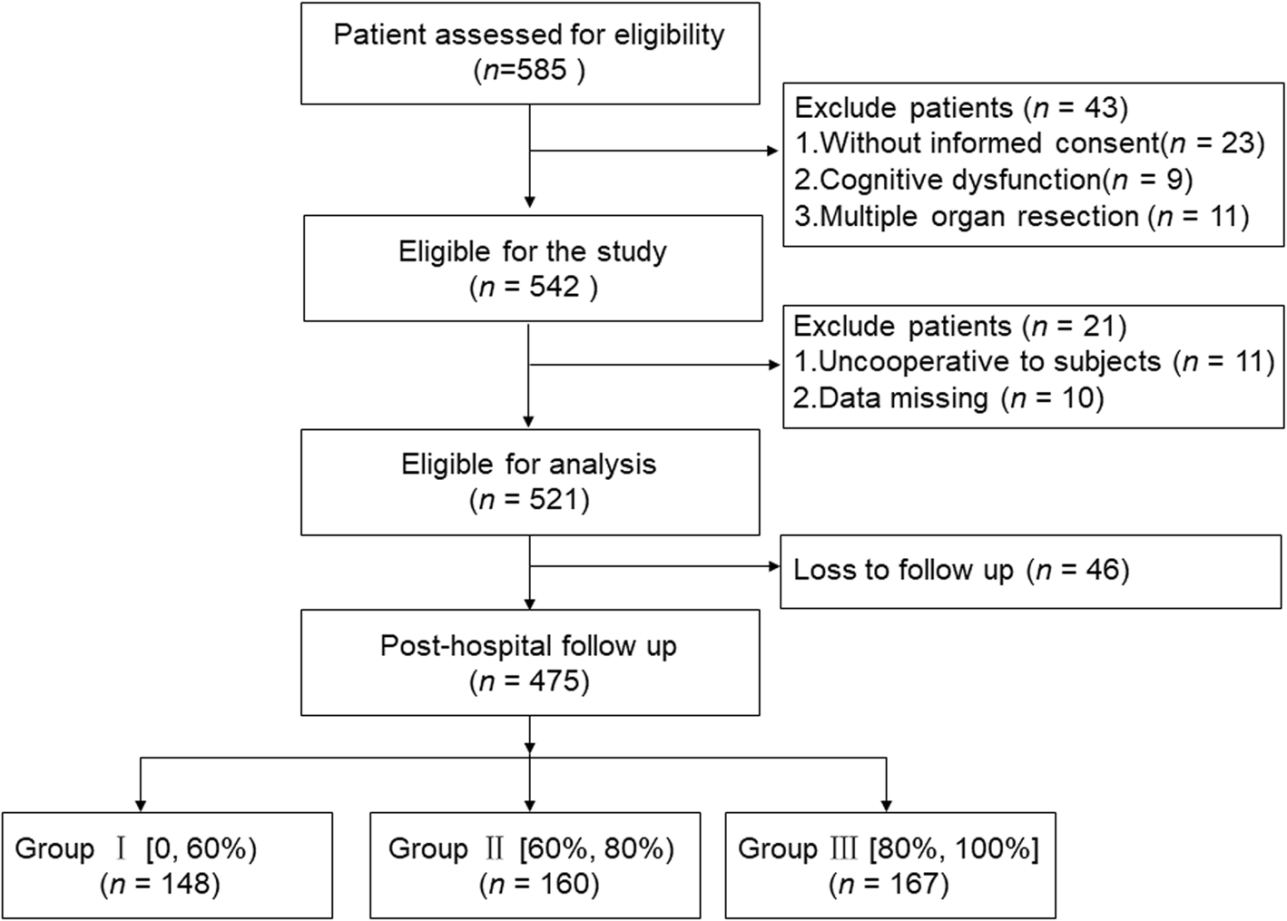
Flowchart of the study participant selection

A total of 585 women were screened for eligibility from February 2019 to December 2020, of whom, 542 were enrolled (showed in Fig. 1). Twenty one women dropped out during the study, leaving 521 women for short-term outcome analyses. Moreover, 46 women were lost during the post-discharge follow-up, allowing for long-term outcomes analyses of 475 women, ranging from 37 to 70 years old, with the median age 49 years old. The overall compliance rate of ERAS protocol modified for gynecological surgery in this study was 80.6%. According to the compliance with the ERAS protocols, women were categorized into three groups: Group I included women with compliance less than 60%; Group II, those with 60 to 80% compliance; and Group III, women with more than 80% compliance. The number of patients in each group was 148, 160, and 167.

No significant differences in patient demographics among the three groups was found, including age, BMI, smoking status, NRS score, ASA status, NYHA status, comorbidity, history of previous lower abdominal surgery and PONV. Baseline assessments and surgical characteristics of the study population are shown in Table 1.

**Table 1:** Baseline Assessments and Surgical Characteristics of the Study Population

Compliance	I[0, 60%) (***N*** = 148)	II[60, 80%) (***N*** = 160)	III[80, 100%] (***N*** = 167)	***P*** value
Age (years^a^), median (IQR)	47.0(44–52)	50.0(43–54)	49.0(47–52)	0.323^△^
BMI (kg/m^2a^), median (IQR)	23.7(22.5–26.6)	25.1(22.0–26.1)	23.4(22.2–25.1)	0.303^△^
Education level above high school, *n*(%)	101(68)	103(64)	98(59)	0.558
Smoking status, *n*(%)				
Never smoked	140(95)	155(97)	158(95)	0.259
Current smoked	2(1)	1(0)	3(2)	0.451*
Former smoked	6(4)	4(3)	6(4)	0.507*
NRS score, *n*(%)				0.398
NRS < 3	98(66)	94(59)	105(63)	
NRS ≥ 3	50(34)	66(41)	62(37)	
Comorbidities, *n*(%)				
Hypertension	28(19)	26(16)	25(15)	0.635
Diabetes	10(7)	6(4)	12(7)	0.363
COPD	4(3)	8(5)	5(3)	0.523*
Ischemic heart disease	3(2)	3(2)	5(3)	0.348*
Asthma	5(3)	2(1)	2(1)	0.120*
NYHA status, *n*(%)				0.558
I	125(84)	144(90)	149(89)	
II	23(16)	16(10)	18(11)	
ASA status, *n*(%)				0.583^△^
I	62(42)	59(37)	75(45)	
II	68(46)	83(52)	77(46)	
III	18(12)	18(11)	15(9)	
Previous lower abdominal surgery, *n*(%)	39(26)	45(28)	40(24)	0.689
Previous PONV, *n*(%)	17(11)	24(15)	32(19)	0.167
Diagnosis, *n*(%)				
Myoma	69(47)	63(39)	74(44)	0.420
Adenomyosis	29(19)	35(22)	38(23)	0.784
Endometriosis	18(12)	22(14)	14(8)	0.291
Cervical dysplasia	7(5)	11(7)	13(8)	0.166*
Other benign	25(17)	29(18)	28(17)	0.939
Surgical approach (laparoscopy/laparotomy)	28/120	11/149	10/157	< 0.001
Preoperative hemoglobin (g/L^a^), median (IQR)	121(101–133)	117(103–138)	122(99–136)	0.356^△^
Preoperative hypokalemia incidence, *n*(%)	44(30)	29(18)	25(15)	0.003
Duration of operation (min^a^), median (IQR)	95(70–125)	105(75–140)	105(80–140)	0.446^△^
Intraoperative blood loss (ml^a^), median (IQR)	50(50–100)	50(50–90)	50(50–100)	0.609^△^
Intraoperative net fluid input (ml^a^), median (IQR)	1600(1100–2000)	1600(1100–1600)	1500(1000–1500)	0.072^△^

*Abbreviations*: *ASA* American Society of Anesthesiologists, *BMI* Body mass index, *COPD* Chronic obstructive pulmonary disease, *IQR* Interquartile range, *NRS* Nutritional risk screening, *NYHA* New York Heart Association, *PONV* Postoperative nausea and vomiting

^a^Continuous variables were described as median (IQR), categorical variables as number of events (*n*)

*Fisher exact test, ^△^Kruskal-Wallis test, all other statistics: Chi-Square test; statistical significance was considered when *P* value **<** 0.05

Furthermore, no clinically significant differences were observed in diagnosis, duration of operation, preoperative hemoglobin or bleeding volume. Significantly, there was a higher proportion of preoperative hypokalemia incidence in the Group I than that in groups II and III (I vs II: 30% vs 18%, *P* = 0.007; I vs III: 30% vs 15%, *P* = 0.002). Meanwhile, the intraoperative net fluid input volume of Group III was lower than that of other groups, but the difference was not statistically significant.

### Postoperative complications

Postoperative complications of the three groups were shown in Table 2. There was a significant decrease in the proportion of patients with any complication between Group I and III (*P* = 0.013). Compared with group I, the incidence of PONV and moderate-to-severe postoperative pain decreased significantly than that in the group III (*P* < 0.001). The incidence of pulmonary infection was 6.1, 6.3, and 1.8% (*P* = 0.044). No statistically significant difference in the other complications among the three groups was observed (*P* > 0.05).

**Table 2:** Comparison of Postoperative Complications and other Perioperative Outcomes among the Groups

Compliance	I[0, 60%) (***N*** = 148)	II[60, 80%) (***N*** = 160)	III[80, 100%] (***N*** = 167)	***P*** value
PONV, n (%)	39(26.3)	40(25.0)	26(15.6)	0.039
Moderate–to–severe postoperative pain, n (%)	25(16.9)	23(14.4)	7(4.2)	< 0.001
DVT, n (%)	1(0.7)	1(0.6)	0(0.0)	0.307*
Surgical site infection, n (%)	5(3.4)	5(3.1)	3(1.8)	0.244*
Pulmonary infection, n (%)	9(6.1)	10(6.3)	3(1.8)	0.044*
Overall complications, n (%)	61(41.2)	61(38.1)	34(20.4)	< 0.001
No–planned re–operation, *n*(%)	0(0)	1(1)	0(0)	0.373*
Readmission, *n*(%)	0(0)	0(0)	0(0)	
Mortality, *n*(%)	0(0)	0(0)	0(0)	
LOS after surgery(d^a^), median (IQR)	5(4–6)	4(3–5)	4(3–4)	0.007^△^
Postoperative hospitalization cost (Euro^a^), median (IQR)	3856(2194)	2591(2170)	2453(2388)	0.134^△^

*Abbreviations*: *DVT* Deep vein thrombosis, *IQR* Interquartile range, *POD* Postoperative day, *PONV* Postoperative nausea and vomiting;

^a^Continuous variables were described as median (IQR), categorical variables as number of events (*n*)

*Fisher exact test, ^△^Kruskal-Wallis test and Mann-Whitney U test was used, all other statistics: Chi-Square test; statistical significance was considered when *P* value **<** 0.05

### QoR-15 scores and other perioperative outcomes

**Fig. 2:**
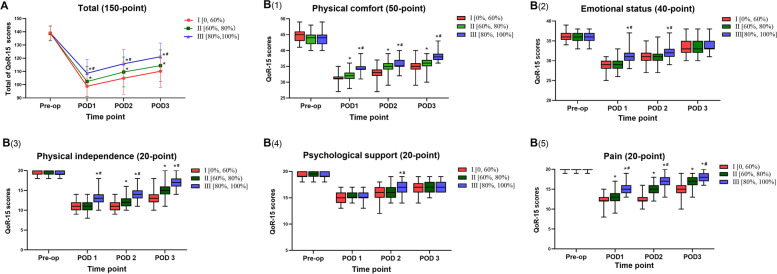
**A** Comparison of total QoR-15 scores in the groups for the pre-operation and the first 3 days after surgery. Range 0–150, higher score indicates better recovery. **B** Comparison of each dimension varies of QoR-15 scores in the groups for the pre-operation and the first 3 days after surgery. Abbreviations: POD, postoperative day; pre-op, pre-operation; QoR-15, 15-item quality of recovery scale. Mann-Whitney U test or repeated measures analysis of variance was used. Compared with group I, *indicated statistical significance (*P* < 0.05); compared with group II, ^#^ indicated statistical significance(*P* < 0.05); and the *P* value was corrected using Bonferroni’s method

No significant difference in the QoR-15 scores among the three groups for the day before operation was found (Fig. 2). Compare with Group I, the total QoR-15 scores in the groups II and III were higher in the first 3 days after surgery (*P <* 0.001). In addition, compare with Group II, the total QoR-15 scores in Group III were higher in the first 3 days after surgery (*P <* 0.001) (Fig. 2A).

Among the five dimensions of the QoR-15, scores of physical comfort (*P* < 0.05), physical independence (*P* < 0.05), and pain dimension (*P* < 0.05) in the group II and III were significantly higher compared to the group I in the first 3 days after surgery; scores of emotional status (*P* < 0.05) in the group III was significantly higher than those in the group I and II in the first 2 days after surgery; scores of psychological support (*P* < 0.05) in the group III were significantly higher compared to the group I and II on POD 2(Fig. 2B).

In this study, no patients developed serious postoperative complications within 30 day after operation (such as ileus, atelectasis, myocardial infarction and bleeding complications), meanwhile no significant differences in the incidence of no-planned re-operation and postoperative hospitalization cost was observed (Table 2). One localized subcutaneous hematoma beneath the incision occurred in one patient and experienced no-planned re-operation, with no patients experiencing re-admission or died during hospitalization and within 30 days after discharge.

### Patient satisfaction

**Fig. 3:**
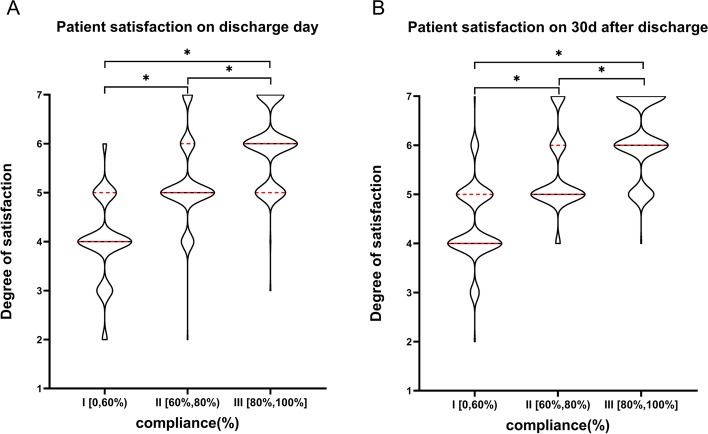
Comparison of patient satisfaction in each group on discharge day (**A**) and the 30 days after discharge (**B**). Range 0–7, higher degree indicates better patient satisfation. Mann-Whitney U test was used. *Indicated statistical significance (*P* < 0.001), and the *P* value was corrected using Bonferroni’s method

Patient satisfaction on discharge day and the 30 days after discharge in each group was shown in Fig. 3. It was significantly higher in the groups II and III than that in the Group I on the discharge day (I vs II: 4(4**–**4) vs 5(5**–**6), *P* < 0.001; I vs III: 4(4**–**4) vs 6(5**–**6), *P* < 0.001; II vs III:5(5**–**6) vs 6(5**–**6), *P* < 0.001). In addition, patient satisfaction was also significantly higher in the groups II and III than that in the Group I on the 30 day after discharge (I vs II: 4(4**–**5) vs 5(5**–**6), *P* < 0.001; I vs III: 4(4**–**5) vs 6(6**–**7), *P* < 0.001; II vs III:5(5**–**6) vs 6(6**–**7), *P* < 0.001).

### Association of complications and compliance with each ERAS component and the predictive nomogram development and validation

The univariable logistic regression analysis was used to analyze the compliance with each ERAS component that might affect the probability of having any postoperative complication. (Table 3). The factors with *P* values more than 0.05 were excluded from multivariate analysis, including no bowel preparation (*P* = 0.573), oral carbohydrate loading (*P* = 0.121), no abdominal drainage (*P* = 0.062), no routine nasogastric tube (*P* = 0.339), postoperative glucose control (*P* = 0.203) and peritoneal drainage (*P* = 0.215). The other factors with *P* values less than 0.05, were further included in the multivariable logistic regression. Finally, five factors with *P* values less than 0.05 in multivariable regression analysis were recruited to construct the prediction model, including minimally invasive surgery (*p* < 0.001), PONV prophylaxis with over 2 antiemetic agents (*p* < 0.001), early mobilization (*p* = 0.031), early oral intake (*p* = 0.012), and early removal of urinary drainage (*p* < 0.001).

**Table 3:** Association of having any postoperative complication within 30d and compliance with each ERAS component

ERAS component	Univariable analysis			Multivariable analysis		
	OR	95% CI	***P***–value	OR	95% CI	***P***–value
Education and counseling	0.26	0.11–0.63	0.003	0.25	0.44–1.42	0.118
Pre–operative optimization	0.50	0.33–0.75	0.001	2.07	0.98–4.40	0.057
No prolonged fasting	2.07	1.09–3.94	0.026	1.04	0.41–2.61	0.938
No pre–anesthetic medication	0.24	0.11–0.49	< 0.001	0.73	0.17–3.11	0.666
Standard anesthetic protocol	0.33	0.21–0.52	< 0.001	0.71	0.25–2.06	0.530
Minimally invasive surgery	0.18	0.09–0.33	< 0.001	0.13	0.05–0.34	< 0.001
Goal–directed fluid therapy	0.49	0.32–0.75	0.001	0.60	0.32–1.11	0.101
Maintenance of normothermia	0.19	0.04–0.98	0.048	0.67	0.03–9.97	0.812
PONV prophylaxis	0.06	0.03–0.12	< 0.001	0.05	0.02–0.12	< 0.001
Multimodal prevention of DVT	0.52	0.35–0.77	0.001	1.08	0.55–2.15	0.824
Avoid salt–water overload	1.89	1.11–3.20	0.019	1.36	0.61–3.01	0.454
Multimodal analgesia	0.38	0.25–0.57	< 0.001	0.97	0.37–2.54	0.963
Early mobilization	0.28	0.19–0.43	< 0.001	0.50	0.27–0.94	0.031
Early oral intake	0.21	0.13–0.32	< 0.001	0.37	0.17–0.81	0.012
Urinary drainage	0.08	0.05–0.13	< 0.001	0.06	0.03–0.12	< 0.001

*Abbreviations*: *CI* Confidence interval, *DVT* Deep vein thrombosis, *ERAS* Enhanced recovery after surgery, *OR* Odds ratio, *PONV* Postoperative nausea and vomiting

**Fig. 4:**
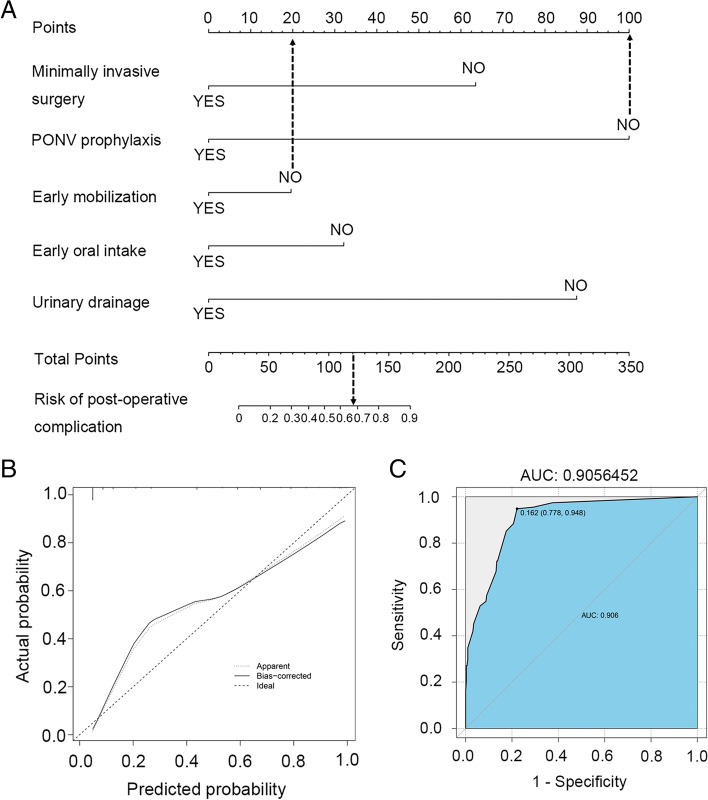
Nomogram to predict risk of postoperative complication. **A** Each factor corresponds to a specific point by drawing a line straight upward to the Points axis. The probability of postoperative complication is the point by drawing a line straight down to the bottom axis from the sum of the points on the Total Points axis. Calibration curve and ROC curve of the nomogram prediction model. **B** Calibration curve. The dashed line represents the ideal fit; the solid line represents the actual fit (**C**) ROC curve. (AUC = 0.906)

The nomogram prediction model was established with 5 independent factors to predict the risk of postoperative complications. As shown in Fig. 4, each factor corresponds to a specific point by drawing a line straight upward to the Points axis. The probability of postoperative complication is the point by drawing a line straight down to the bottom axis from the sum of the points on the Total Points axis. For example, according to the model, a woman who underwent minimally invasive surgery (0 point) treated with early oral intake (0 point) and early urinary drainage (0 point), but not with PONV prophylaxis (100 point) and early mobilization (20 point) with about 67% incidence of postoperative complications. The validation of the the nomogram prediction model was based on calibration and discrimination. The calibration curve demonstrated a good calibration because the actual line was not significantly deviated from the ideal line. The result of ROC curve showed an excellent discrimination with a high AUC value (0.906) and sensitivity (0.948).

## Discussion

Implementation of the ERAS pathway remains an ongoing challenge in clinical practice which requires the engagement of nursing team, surgical team, and patients. Individual ERAS components are implemented at different stages during the patient**’**s hospitalization, which may adversely influence compliance. Furthermore, implementation of ERAS pathway is associated with a shift in clinical routines, from old practices to new pathway. This study is the first study to our knowledge determining the effect of the compliance of patients to an ERAS protocol after benign hysterectomy on outcomes with the QoR-15 scales and patient satisfaction. Our current study indicated that an overall compliance rate of 80.6% for ERAS in patients undergoing hysterectomy. Similarly, a recent observational study reported a mean compliance rate of 77% after the ERAS program was implemented in gynecologic oncology [29]. Consistent with other studies, improved adherence to the ERAS protocols improved clinical rehabilitation [4, 5, 30], we found patients with compliance over 80% had a significant reduction in postoperative complications and length of stay after surgery. Increased compliance was also associated with higher patient satisfaction and QoR-15 scores. The difference in the incidence of postoperative complications within 30 days between the lowest and highest compliance groups was 20.8%.

Most previous studies evaluating postoperative recovery have focused on physiological parameters such as LOS and morbidity [22, 31–34]. As the recovery process is complex and encompasses the multiple dimensions of physical [35], emotional and social health, patients’ reported outcome are essential to evaluate the quality of recovery, measuring any aspect of a patient’s health status with information derived directly from the patient. Patient’s health status was measured with the QoR-15 scale and patient satisfaction. Meanwhile Chinese version QoR-15 has good reliability, validity, clinical acceptability and feasibility [23, 24, 36].

We found the QoR-15 scores and patient satisfaction were significantly higher in the higher compliance group, which can be explained by the reduction pain and improvement mood during the early postoperative period. In the ERAS protocols, preoperative information education, no prolonged fasting, no bowel preparation and oral carbohydrate loading can relieve the patient discomfort such as anxiety, hungry and thirsty. In addition to the above advantages, it also increases patient comfort and physical independence by reducing the incidence of postoperative nausea and vomiting, relieving pain and accelerating the recovery. The increase in patient satisfaction after multimodal analgesic approaches may be a significant benefit, along with a reduction in postoperative complications. In order to avoid the interference of other factors on the results, we really scored the patients, and we chose to conduct the satisfaction survey of the patients on the day of discharge and 30 days after discharge.

Suggestions for improving compliance include the use of a dedicated wards, specific personnel, effective education and training, and regular inspections [37]. The lack of repeated education on ERAS protocols has a significant influence on compliance, as well as analyzing the obstacles and catalysts to implementation and compliance. Every participant needs to know the implementation of ERAS protocols can be challenging, but ultimately rewarding [38].

No serious complications were observed in this study. However, due to our relatively small trial size, which limited our ability to judge the frequency of rare but potentially serious events. PONV is still the main problem affecting the early postoperative feeding and activity of patients. General anesthesia combined with TAP block and multimodal postoperative analgesia pathway could reduce the use of opioids, meanwhile reduce the occurrence of PONV [39]. At present, the relationship between major complications and compliance with ERAS remains the focus of research [40].

We also noted that the incidence of preoperative hypokalemia was higher in patients with < 60% compliance, it may be attributed to the no prolonged fasting, as well as insulin resistance associated with oral carbohydrate intake, which deserved further research. Several limitations of the current study need to be addressed. First, a randomized controlled trial should be the preferred option, yet now the existing evidence on ERAS protocols; we considered it unethical to conduct a randomized controlled trial. However, the relatively large sample size, the fixed ERAS MDT and the prospective data collection by a dedicated nurse are strengths of this study. Although most data were entered prospectively, analysis was done retrospectively and results should be interpreted as such. Further researches into the ERAS pathway are needed, including using validated international classification systems such as Clavien-Dindo, and evaluation of additional patient-related outcomes such as patient experience of the process and longer-term consequences. In addition, the predictive nomogram model without a subsequent validation study, it cannot be extrapolated. And the validation and optimization of current model needed to be performed in future study.

## Conclusion

In summary, better compliance to the ERAS protocols modified for gynecological surgery is crucial to postoperative quality of recovery after benign hysterectomy, decrease the overall complication rate and improve patient experience without a significant increase in readmission and mortality. In this study in particular, minimally invasive surgery, PONV prophylaxis, early mobilization, early oral intake, and early removal of urinary drainage were associated with a lower complication rate after benign hysterectomy.

## Supplementary Information

**Additional file 1.** ERAS protocol used in our department.

**Additional file 2.** QoR-15 questionnaire used in the study.

**Additional file 3.** Definition of postoperative complications in the study.

## Data Availability

The datasets used and analyzed in this study is available from the corresponding author on reasonable request.

## Abbreviations

ASA: American Society of Anesthesiologists
BMI: Body mass index
CI: Confidence interval
COPD: Chronic obstructive pulmonary disease
DVT: Deep vein thrombosis
ERAS: Enhanced recovery after surgery
NRS: Nutritional risk screening
MDT: Multi-disciplinary team
NYHA: New York Heart Association
POD: Postoperative day
PONV: Postoperative nausea and vomiting
QoR-15: 15-item quality of recovery scale
