# Supplementary material for: Impact of enhanced recovery after surgery protocol compliance on patients’ outcome in benign hysterectomy and establishment of a predictive nomogram model
Source: BMC Anesthesiol. 2021 Nov 22;21:289. doi: 10.1186/s12871-021-01509-0 (PMC8607678; doi:10.1186/s12871-021-01509-0)
Supplement: Supplementary file 1 — Additional file 1. ERAS protocol used in our department. [file 12871_2021_1509_MOESM1_ESM.pdf]

**Additional file 1**

**ERAS protocol used in our department (22 items)**

| | |
|---|---|
| Pre-operative | Preoperative information education and counseling |
| | Pre-operative optimization (Cardiopulmonary function evaluation and optimization, Nutritional assessment and enteral nutrition support, smoking and alcohol consumption should be stopped 4 weeks before surgery ) |
| | No prolonged fasting (6-8 hours for solid food, 2 hours for clear liquids) |
| | No bowel preparation |
| | Pre-operative antibiotics (Intravenous cefoxitin 1.5 g or ceftriaxone 1 g 30 minutes before incision) |
| | Pre-operative Oral carbohydrate loading(intake of 400 ml 10% glucose solution: up to 2-3 hours before the induction of anesthesia ) |
| | No pre-anesthetic medication |
| Intra-operative | Standard anesthetic protocol (General anesthesia with rapid short-acting agents combined with TAP block and lung-protective ventilatory strategy) |
| | Minimally invasive surgery (Laparoscopic or robotic surgery) |
| | No abdominal drainage |
| | Goal-directed fluid therapy by anesthetic team with a focus on avoiding fluid overload |
| | Maintenance of normothermia |
| | PONV prophylaxis (with >2 antiemetic agents) |
| | Multimodal prevention of DVT (physical prophylaxis combined with low molecular weight heparin administration) |
| | No routine nasogastric tube |
| Post-operative | Post-operative glucose control |
| | Avoid salt–water overload |
| | Multimodal analgesia(PCIA, TAP, NSAIDs, COX-2 inhibitor) |
| | Early mobilization (out-of-bed activity for 2 hours on the first postoperative day and 4-6 hours from the second postoperative day to discharge) |
| | Early oral intake (drink water 2 hours after surgery, oral nutritional supplements |

| | |
|---|---|
| | on the first day after surgery, semisolid diet on the second day after surgery) |
| | Peritoneal drainage (Early removal of drainage tubes within three days after surgery) |
| | Urinary drainage (Early removal of urinary catheter within 24 hours) |

Either implementation of new elements or adjustment of existing perioperative care elements with institution of standardized perioperative order sets and education of house staff.

Abbreviations:COX: cyclooxyganese; DVT: deep vein thrombosis; PONV: postoperative nausea and vomiting; PCIA: patient controlled intravenous analgesia; TAP: transversus abdominis plane; NSAIDs: nonsteroidal anti-inflammatory drugs.
