# Supplementary material for: Impact of enhanced recovery after surgery protocol compliance on patients’ outcome in benign hysterectomy and establishment of a predictive nomogram model
Source: BMC Anesthesiol. 2021 Nov 22;21:289. doi: 10.1186/s12871-021-01509-0 (PMC8607678; doi:10.1186/s12871-021-01509-0)
Supplement: Supplementary file 2 — Additional file 2. QoR-15 questionnaire used in the study. [file 12871_2021_1509_MOESM2_ESM.pdf]

Additional file 2

QoR-15 patient survey

Date: _________ **CRF No.______**

Preoperative ☐ **postoperative POD1☐ POD2☐ POD3☐**

PART A

How have you been feeling in the last 24 hours?

(0 to 10 ,where: 0= none of the time [poor] and 10 =all of the time [excellent])

**1. Able to breathe easily**	**0 1 2 3 4 5 6 7 8 9 10**
**2. Been able to enjoy food**	**0 1 2 3 4 5 6 7 8 9 10**
**3. Feeling rested**	**0 1 2 3 4 5 6 7 8 9 10**
**4. Have had a good sleep**	**0 1 2 3 4 5 6 7 8 9 10**
**5. Able to look after personal toilet and hygiene unaided**	**0 1 2 3 4 5 6 7 8 9 10**
**6. Able to communicate with family or friends**	**0 1 2 3 4 5 6 7 8 9 10**
**7. Getting support from hospital doctors and nurses**	**0 1 2 3 4 5 6 7 8 9 10**
**8. Able to return to work or usual home activities**	**0 1 2 3 4 5 6 7 8 9 10**
**9. Feeling comfortable and in control**	**0 1 2 3 4 5 6 7 8 9 10**
**10. Having a feeling of general well-being**	**0 1 2 3 4 5 6 7 8 9 10**

PART B

Have you had any of the following in the last 24 hours?

(0 to 10 ,where: 0= none of the time [excellent] and 10 =all of the time [poor])

**11. Moderate pain**	**0 1 2 3 4 5 6 7 8 9 10**
**12. Severe pain**	**0 1 2 3 4 5 6 7 8 9 10**

**13. Nausea or vomiting**	**0 1 2 3 4 5 6 7 8 9 10**
**14. Feeling worried or anxious**	**0 1 2 3 4 5 6 7 8 9 10**
**15. Feeling sad or depressed**	**0 1 2 3 4 5 6 7 8 9 10**
