# Supplementary material for: Impact of enhanced recovery after surgery protocol compliance on patients’ outcome in benign hysterectomy and establishment of a predictive nomogram model
Source: BMC Anesthesiol. 2021 Nov 22;21:289. doi: 10.1186/s12871-021-01509-0 (PMC8607678; doi:10.1186/s12871-021-01509-0)
Supplement: Supplementary file 3 — Additional file 3. Definition of postoperative complications in the study. [file 12871_2021_1509_MOESM3_ESM.pdf]

## Additional file 3

### Definition of postoperative complications

| Complication | Definition |
|---|---|
| Postoperative nausea and vomiting(PONV) | Postoperative nausea and vomiting within 72 hours after surgery. |
| Moderate-to-severe postoperative pain | VAS≥4 was considered to identify patients with postoperative pain of moderate-to-severe intensity. |
| Deep vein thrombosis (DVT) | Postoperative ultrasonography revealed a new blood clot or thrombus in the venous within 30 days after surgery. |
| Surgical site infection(SSI) | Infection occurs within 30 days after surgery and involves only skin and subcutaneous tissue of the incision, and the patient has at least one of the following: purulent drainage from the superficial incision; organisms isolated from an aseptically obtained culture of fluid or tissue from the superficial incision; at least one of the following symptoms or signs of infection: pain or tenderness, localised swelling, redness or heat; diagnosis of an incisional surgical site infection by a surgeon or attending physician. |
| Pneumonia/pulmonary infection | Chest radiograph with at least one of the following: infiltrates, consolidation, cavitation; and at least one of the following: fever (>38°C) with no other recognized cause, white cell count $>12\times10^9$ /l or $<4\times10^9$ /l, >70years old with altered mental status with no other recognized cause; and at least two of the following: purulent sputum or increased respiratory secretions, cough or dyspnoea or tachypnoea, rales or bronchial breath sounds, worsening gas exchange. |
| Paralytic ileus | Failure to tolerate solid food or defecate for three or more days after surgery. |
| Atelectasis | Lung opacification with a shift of the mediastinum, hilum or hemidiaphragm toward the affected area, and compensatory overexpansion in the adjacent non-atelectatic area. |

Abbreviations: DVT: deep vein thrombosis; PONV: postoperative nausea and vomiting; SSI:Surgical site infection.

The postoperative complications were defined by the guidelines for European perioperative clinical outcome definitions[1].

# References

1. Jammer I, Wickboldt N, Sander M, Smith A, Schultz MJ, Pelosi P, Leva B, Rhodes A, Hoeft A, Walder B, Chew MS, Pearse RM, European Society of Anaesthesiology (ESA) and the European Society of Intensive Care Medicine (ESICM), European Society of Anaesthesiology, European Society of Intensive Care Medicine: Standards for definitions and use of outcome measures for clinical effectiveness research in perioperative medicine: European Perioperative Clinical Outcome (EPCO) definitions: a statement from the ESA-ESICM joint taskforce on perioperative outcome measures. *Eur J Anaesthesiol* 2015, 32:88-105.
